# Characteristics of and Influencing Factors of Hydrochemistry and Carbon/Nitrogen Variation in the Huangzhouhe River Basin, a World Natural Heritage Site

**DOI:** 10.3390/ijerph182413169

**Published:** 2021-12-14

**Authors:** Chenpeng Hu, Ziqi Liu, Kangning Xiong, Xiaoxi Lyu, Yuan Li, Renkai Zhang

**Affiliations:** 1 School of Karst Science, Guizhou Normal University, Guiyang 550001, China; hcpmax@163.com (C.H.); 201511004@gznu.edu.cn (Z.L.); karstlv@gznu.edu.cn (X.L.); liyuan7pro@163.com (Y.L.); zhangrk9818@163.com (R.Z.); 2 State Engineering Technology Institute for Karst Desertification Control, Guiyang 550001, China

**Keywords:** water chemistry, dolomite, chemical weathering, dissolved organic carbon, total nitrogen, Huangzhouhe River

## Abstract

In karst areas, the characteristics of water chemistry and carbon and nitrogen are of great significance to basic research. The contents of Ca^2+^, Mg^2+^, K^+^, Na^+^, HCO_3_^−^, SO_4_^2−^, NO_3_^−^, Cl^−^, dissolved organic carbon (DOC), and total nitrogen (TN) in water samples from 18 rivers and 14 springs in the Huangzhouhe River Basin were determined. The results showed that the water chemistry type in the Huangzhouhe River Basin is HCO_3_-Ca-Mg. The chemical composition is mainly affected by dolomite weathering and also by ion exchange and other human activities. The river and spring DIC remain at the same level in the upper and middle reaches and decrease in the lower reaches. The NO_3_-N and TN of river water and TN of spring water increase in the middle reaches, while NO_3_-N of spring water decreases in the lower reaches. The DOC in the basin increases with the increase of SO_4_^2−^ and Cl^−^, mainly due to the human influence of agricultural and domestic sewage. In the basin, the NO_3_-N and TN in spring water are larger, and the DOC in river water is larger, mainly because there are more phytoplankton and human activities in the river water. The carbon and nitrogen in the Huangzhouhe River Basin are mainly HCO_3_^−^ and NO_3_^−^ ions. The evaluation of pH, Cl^−^, NO_3_-N, SO_4_^2^^−^, and TDS shows that the water quality is good and the ecological environment is good.

## 1. Introduction

The input of terrestrial erosion material from rivers to the ocean is a key part of the biogeochemical cycle. River chemistry is influenced by many natural geochemical processes such as climate, structure, weathering, and vegetation cover [1,2,3,4]; lithology (rock weathering) is the dominant factor in these processes because rock is the main source of dissolved load in river water [5,6]. The content and distribution characteristics of ions in rivers are influenced and controlled by many factors such as precipitation, temperature, topography, altitude, recharge type, runoff and discharge processes, land use type, and surface lithology. In the Garhwal Bhagirathi watershed, chemical parameters, anions, and cations are related to geomorphological parameters and land cover parameters, and they change with the season [7]. The characteristics of water chemistry and their variation can indicate the main ion sources of the water body and reflect the influence of human activities on water environments. The inorganic carbon dissolution load in river water comes from different sources: rock dissolution, precipitation, and human input [8].

River DOC and TN mainly come from soil leaching [9,10], complex biological processes (e.g., production and release of bacteria and phytoplankton, and feeding of zooplankton) [11,12,13], and human input [14]. The DOC content affects aquatic communities [15] and facilitates heavy metal migration in river water by forming organic complexes [16,17], and may lead to enrichment with toxic metals [18]. The DOC is an important part of the global carbon cycle, and is related to the environment and climate change [19,20,21]. Dissolved organic carbon, as an indicator of organic matter levels, can reflect the extent of organic pollution in rivers and is closely related to water quality [22,23]. It is well known that Cl^−^, dissolved inorganic nitrogen (DIN), and SO_4_^2−^ are mostly derived from human inputs [8,24]. Abnormal changes in climate and temperature also impact carbon and nitrogen species. For example, bacterial communities in Poyang Lake are sensitive to DOC and NO_3_^−^ contents in the dry season [25]. On the one hand, waste input into rivers affects the river’s hydrochemical properties and may interfere with natural geochemical processes and accelerate weathering processes [26,27]. On the other hand, river carbon and nitrogen concentrations can be directly increased [28,29,30].

In the hydrological cycle, surface water and groundwater are interdependent, mutually restricted, and independent. For example, the groundwater system of Abadir Farm is invaded by shallow groundwater and Basaka Lake [31]. Dinka [32] found that when surface water and groundwater are polluted, the upper freshwater layer is exchanged with the deeper water, and the frequent exchange of groundwater and river water increases the risk of water pollution. The high content of organic carbon in wastewater can lead to a large amount of microbial growth, making groundwater unsuitable for residential use. The variation in the characteristics of water chemistry and carbon and nitrogen contents not only reflects the ecological evolution characteristics of the region, but also affects the ecological security of the downstream regions.

In the carbonate regions of the world, dolomite is widely distributed as limestone. China is dominated by limestone, but dolomite, dolomitic tuff, and ash dolomite account for 25.7% of the total distribution of carbonate rocks in China. Most research in China has focused on limestone karst areas, with less research on dolomite karst areas. Large areas of dolomite are also exposed in some other provinces in China, mainly as grey dolomite or dolomite interbedded with limestone; pure dolomite up to 1100 m thick is only found in Shibing, Guizhou [33]. Through the study of the temporal and spatial dynamics of the contents of anions and cations in the Huangzhouhe River Basin, we revealed the hydrogeochemical characteristics of typical subtropical dolomite basins in this study. The objectives of this study were to (1) analyze the hydrochemical characteristics of river water and spring water in the basin, (2) determine the variation in water C and N concentrations and their relationship, and (3) clarify the effects of human input on water chemistry and water quality.

## 2. Materials and Methods

### 2.1. Study Area

**Figure 1:**
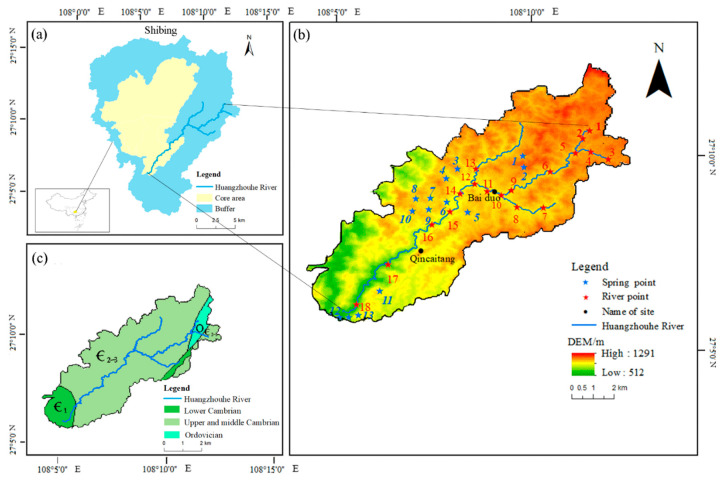
Location of sampling sites in the Huangzhouhe River Basin. (**a**) Buffer and core areas of the Shingbing River. (**b**)The Huangzhouhe River Basin and sampling sites. (**c**) Stratigraphy of the Huangzhouhe River Basin.

The Huangzhouhe River Basin in Guizhou Province is located in a World Natural Heritage Protection Area (Figure 1). It is a complete and typical dolomite karst basin, and is a natural site for studying the weathering of dolomite rocks. In the Huangzhouhe River Basin, limestone is exposed in 8% of the area and dolomite is exposed in 92% of the area. The exposed strata are the Cambrian Gaotai, Loushanguan, and Shilengshui formations, and a small amount is the Qingxudong formation. It flows from the eastern heritage site buffer to the southwest core area, the upper part is a wide valley. Above the Baiduo is the upstream, above the Qingcaitang is the midstream, and below is the downstream. A dolomite landform developed strongly in the basin, and the landform is broken. Due to river-cutting and erosion, the area developed into a typical deep subtropical karst gorge landform. The Huangzhouhe River Basin has a subtropical humid monsoon climate, with rainy and hot periods and abundant precipitation. The annual average temperature is 14–16 °C, the annual rainfall is 1060–1200 mm, the annual average discharge is 4.2 m^3^/s, and the annual average evaporation is 1078.42 mm. The topography of Shibing Karst World Natural Heritage Site in which the Huangzhouhe River is located high in the northwest and low in the southeast, with a core area of 10,280 hm^2^, a buffer area of 18,015 hm^2^, and a total area of 28,295 hm^2^ [33]. The study area is a World Natural Heritage Site. It has a pure natural ecological environment, mainly vegetated by trees and shrubs. The middle and upper reaches of the Huangzhouhe River are located in the buffer zone, with a high population density and diverse land use types, mainly including dry arable land, paddy field, woodland, and shrub forest, where the ecological environment is disturbed by human beings. There is the potential for large amounts of pollutants to flow from the buffer zone into the core area, thereby impacting the ecological health of the site. Therefore, it is of considerable value to study the hydrochemistry, carbon and nitrogen characteristics, and their influencing factors on the Huangzhouhe River for the protection of world natural heritage.

### 2.2. Water Sampling and Analysis

The sampling was conducted in January and July 2021, taking the Huangzhouhe River basin as a whole and collecting 18 river samples from downstream to upstream, tributaries and main streams, and 14 spring samples within the basin, for a total of 32 samples. The samples were washed with a 1 mol·L^−1^ solution of analytically pure HNO_3_, then rinsed clean with pure water and dried to ensure that the ions in the water sample did not adhere to the bottle wall. The collected samples were filtered on the same day (0.45 μm Millipore membrane), and the filtered samples were packed into different pretreatment polyethylene bottles. Cationic polyethylene bottles were acidified on site with 1:1 pure nitric acid to pH < 2; the contents of the anionic polyethylene bottles, and TN and DOC polyethylene bottles were added directly to the filtered water samples, and the polyethylene bottles were sealed with sealing film. The collected samples were stored at low temperature in the field in a refrigerated box and quickly sent to the laboratory.

The water temperature (°C), pH, and conductivity (EC, μS·cm^−1^) of the dripping water were tested in the field with a HQ40d portable water quality analyzer (HACH, Loveland, CO, USA), and the HCO_3_^−^ (mmol·L^−1^) concentration of the water samples was determined through titration, with the value recorded separately with an alkalinity meter (Merck, Germany). The concentrations of anions (Cl^−^, NO_3_^−^, and SO_4_^2−^) were determined by ion chromatography (IC, DX-120, Dionex, Germany). An Optima-2100DV full-spectrum, direct-reading ICP-OES (Perkin-Elmer, USA) was used to determine the concentrations of K^+^, Na^+^, Ca^2+^, and Mg^2+^ in dripping water samples, having a detection accuracy of 0.001 mg·L^−1^ and a relative standard deviation of <2%. The detection limits for all ions were less than 0.1 mg/L. DOC and TN concentrations were determined using a Shimadzu TOC-VCPH total organic carbon analyzer with a relative standard deviation of <1.5%. Each sample value represents the average of two consecutive measurements. The measurement error was less than 1%.

### 2.3. Data Processing

We used IBM SPSS Statistics 26 (IBM, Armonk, NY, USA) and Excel (Microsoft Corporation, Albuquerque, NM, USA) to complete the statistical analysis, and Aq-QA (version 1.1) water chemistry analysis software (RockWare, Inc., Golden, CO, USA) and Origin2021b for plotting (OriginLab. Northampton, MA, USA).

## 3. Results

### 3.1. Physicochemical Parameters and Hydrochemical Type

The water samples in the Huangzhouhe River Basin are generally weakly alkaline, with a pH between 6.98 and 8.99. The pH of the river during the rainy season is slightly higher than during the dry season. The TDS is 202.30–488.50 mg·L^−1^, with an average of 338.4 mg·L^−1^, in the rainy season and 348.58 mg·L^−1^ in the dry season. Table 1 shows that the ion content in the rainy season is slightly higher than that in the dry season.

**Figure 2:**
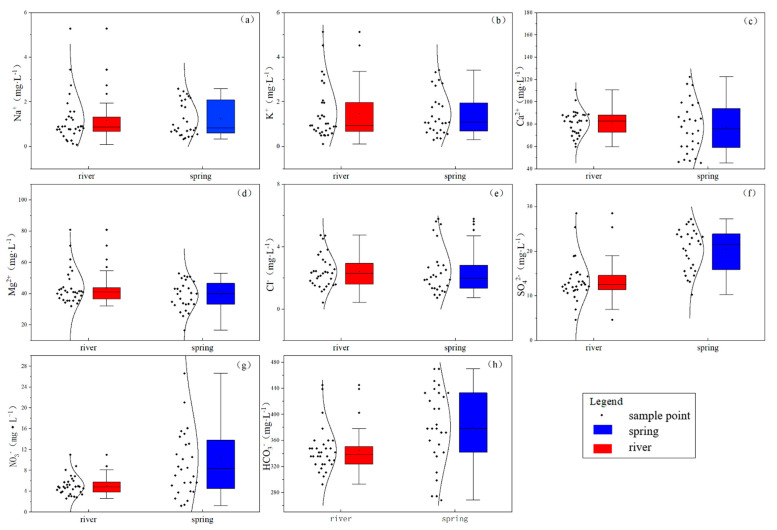
Ion box diagram for the water samples: (**a**–**h**) comparison of ion contents between river water and spring water.

Ca^2+^, Mg^2+^, and HCO_3_^−^ are the dominant ions in the Huangzhouhe River Basin (Figure 2). The decreasing order of cations in the river is Ca^2+^ > Mg^2+^ > K^+^ > Na^+^, with Ca^2+^ and Mg^2+^ accounting for about 97.93% of the cations. The decreasing order of anion concentration is HCO_3_^−^ > SO_4_^2−^ > NO_3_^−^ > Cl^−^, with HCO_3_^−^ accounting for about 94.31%. The cations in the spring are Ca^2+^ > Mg^2+^ > K^+^ > Na^+^, with Ca^2+^ and Mg^2+^ accounting for about 97.83% of the cations. The anion concentration is HCO_3_^−^ > SO_4_^2−^ > NO_3_^−^ > Cl^−^, with HCO_3_^−^ accounting for about 92.05% of the total. The box plot in Figure 2 shows that the Na^+^, K^+^, and Cl^−^ contents in the river water are roughly the same as those in groundwater, so it can be inferred that atmospheric precipitation is the main source of recharge. The Ca^2+^ and Mg^2+^ contents in river water are slightly higher than those in spring water, and the concentration of HCO_3_^−^ in spring water is higher than that in river water, which is related to the longer time of water–rock interaction in spring water. pH and SO_4_^2−^ and NO_3_^−^ contents in spring water are also higher than those in river water.

**Figure 3:**
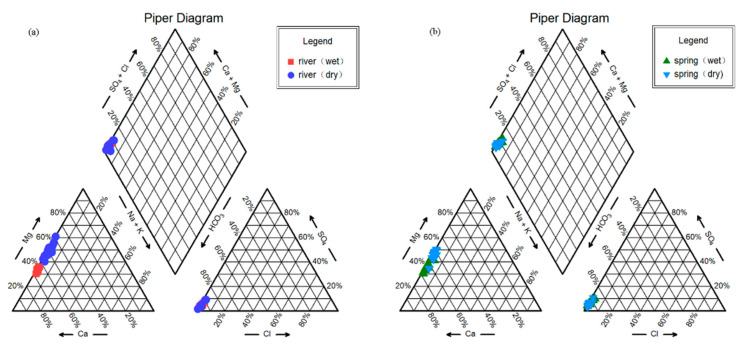
Chemistry Piper diagram of the river (**a**) and spring (**b**) water in the Huangzhouhe River Basin.

The Piper trilinear map, which is divided into nine zones according to ion content, assigns different positions in the diamond to different water chemistries, which can objectively reflect the water chemistry of the region. The trilinear diagram shows that surface water and groundwater have basically the same ion positions, with all having a carbonic acid content of more than 80%, calcium and magnesium contents in the range of 30% to 70%, and the sum of carbonic acid and calcium and magnesium contents accounting for more than 60%, indicating the same water–rock action. The water chemistry type is mainly HCO_3_-Ca-Mg because the study area is controlled by karst area carbonate rocks (Figure 3). After long-term water–rock interaction, groundwater is more concentrated in the lower left corner of the Piper three-line diagram, which is consistent with the results of Nandong karst water in December [34].

### 3.2. C and N Types and Distribution

The DIC in natural water bodies is mainly derived from CO_2_ input from air and soil and the dissolution or precipitation of carbonate rocks [35]. Within the Huangzhouhe River Basin, which is mainly subject to carbonate rock weathering, DIC is mainly derived from dolomite weathering. The DIC in the water column occurs mainly in the form of HCO_3_^−^, CO_3_^2−^, CO_2_, and H_2_CO_3_. HCO_3_^−^ is highly significantly positively correlated with DIC, accounting for 80.39% of the DIC [36]. In this paper, the HCO_3_^−^ content is used to represent the DIC. The TN in a river is input through three main pathways: external input, internal organic matter cycling, and substrate release [37]. In springs, TN is mainly provided from rainfall and overlying vegetation activities.

Table 2 shows the variation law of the concentrations of various forms of carbon and nitrogen concentration in the Huangzhouhe River Basin. In the dry season, the TN content of the river from upstream to downstream is 0.62~1.91 mg·L^−1^, the NO_3_-N content is 0.49~1.19 mg·L^−1^, the DIC content is 305~445.3 mg·L^−1^, and the DOC content is 2.04~4.52 mg·L^−1^. The spring water TN content is 0.29~2.63 mg·L^−1^, the NO_3_-N content is 0.15~1.92 mg·L^−1^, the DOC content is 1.48~4.85 mg·L^−1^, and the DIC content is 274.5~469.7 mg·L^−1^. In the rainy season, the TN content of the river from upstream to downstream is 1.22~3.23 mg·L^−1^, the NO_3_-N content is 0.77~1.29 mg·L^−1^, the DIC content is 292.87~378.2 mg·L^−1^, and the DOC content is 2.57~4.81 mg·L^−1^. Spring water’s TN content is 1.62~3.78 mg·L^−1^, its NO_3_-N content is 0.89~2.51 mg·L^−1^, the DOC content is 1.66~3.36 mg·L^−1^, and the DIC content is 274.5~469.7 mg·L^−1^. The NO_3_-N and TN contents in river water are higher in the middle reaches, DIC content is slightly lower in the downstream area, and DOC content decreases along the river from the upper to lower reaches. In spring water, NO_3_-N, TN, and DIC contents are lower in the downstream area, and the DOC content is higher in the middle reaches. In general, the carbon and nitrogen contents of spring water are higher than those in river water.

## 4. Discussion

### 4.1. Analysis of Major Ion Sources

**Figure 4:**
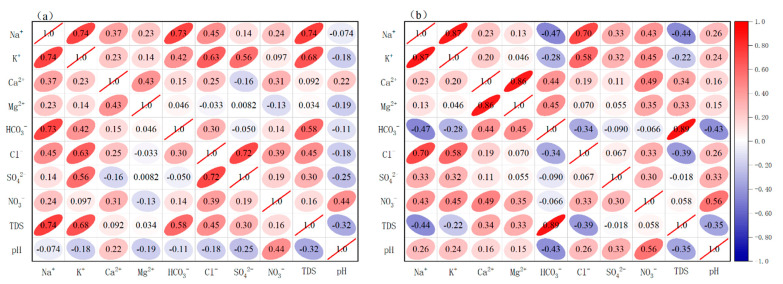
Ion correlation in the water samples: (**a**) river water ion correlations and (**b**) spring water ion correlations.

Correlation analysis between anions and cations can reflect the material origin of the ions and the characteristics of the chemical reactions they underwent. Ions with stronger correlations may be from the same material or may have undergone the same chemical reaction process. The Ca^2+^ and Mg^2+^ in the river water in the basin are strongly correlated, as are the K^+^ and Na^+^, and Cl^−^ and SO_4_^2−^ (Figure 4a), which indicates that Ca^2+^ and Mg^2+^, Cl^−^ and SO_4_^2−^, and K^+^ and Na+ came from different substances or underwent the same chemical process. The analysis is provided below. In the basin spring water, TDS and HCO_3_^−^ are extremely correlated (Figure 4b) and show strong correlation with Ca^2+^ and Mg^2+^, where Ca^2+^, Mg^2+^ and HCO_3_^−^ are all major ions. HCO_3_^−^ correlates with both Ca^2+^ and Mg^2+^, indicating a similar origin. In the basin water samples, Ca^2+^ shows an extremely strong correlation with Mg^2+^ and TDS with HCO_3_^−^, indicating that carbonate rocks are the main factor controlling water chemistry in the study area.

**Figure 5:**
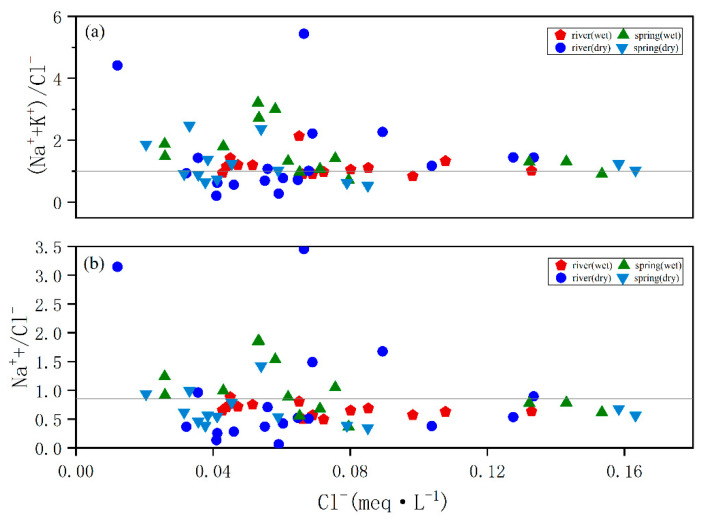
Ion ratio in water samples: (**a**) Na^+^/Cl^−^ and (**b**) (Na^+^ + K^+^)/Cl^−^ of the river and spring waters.

Atmospheric precipitation, evaporite dissolution, rock weathering, and anthropogenic input are the sources of ions in the water column. The source and chemical reaction characteristics can be inferred from the ion content, species, and ratio in the water column [38,39]. The main sources of K^+^ and Na^+^ in the water column are atmospheric precipitation, evaporite minerals, and silicate minerals. Numerous studies have shown that the average Na^+^/Cl^−^ value in atmospheric precipitation and seawater is about 0.86, and when evaporite is dissolved without other influences, the (Na^+^ + K^+^)/Cl^−^ ratio is about one. In Figure 5, water samples are evenly distributed on both sides of the straight line, but there is no evaporite in the study area, indicating other influences, which will be analyzed below. The ratio of (Na^+^ + K^+^)/Cl^−^ and Na^+^/Cl^−^ in the water samples in the rainy season is larger than that in the dry season because the water is more affected by humans in summer. In Figure 4, the river water Na^+^, K^+^, and Cl^−^ contents are highly correlated, indicating that atmospheric precipitation is the main source of Na^+^, K^+^, and Cl^−^, being also affected by humans.

When only dolomite is dissolved, the ratio of γHCO_3_^−^ to γCa^2+^ is close to 4:1, the ratio of γHCO_3_^−^ to γMg^2+^ is close to 4:1, and the ratio of γCa^2+^ to γMg^2+^ is close to 1:1. When only calcite is dissolved in groundwater, the ratio of γHCO_3_^−^ to γCa^2+^ is close to 1:2. Since there is no Mg in calcite (CaCO_3_), the content of Mg^2+^ in groundwater is low and can be ignored. When calcite (CaCO_3_) and dolomite (CaMg(CO_3_)_2_) dissolve simultaneously in karst groundwater, the ratio of γHCO_3_^−^ to γCa^2+^ is close to 3:1, and the ratio of γHCO_3_^−^ to γMg^2+^ is close to 6:1. Correlation analysis showed that Mg^2+^, Ca^2+^ and HCO_3_^−^ are strongly correlated, indicating strong homology.

**Figure 6:**
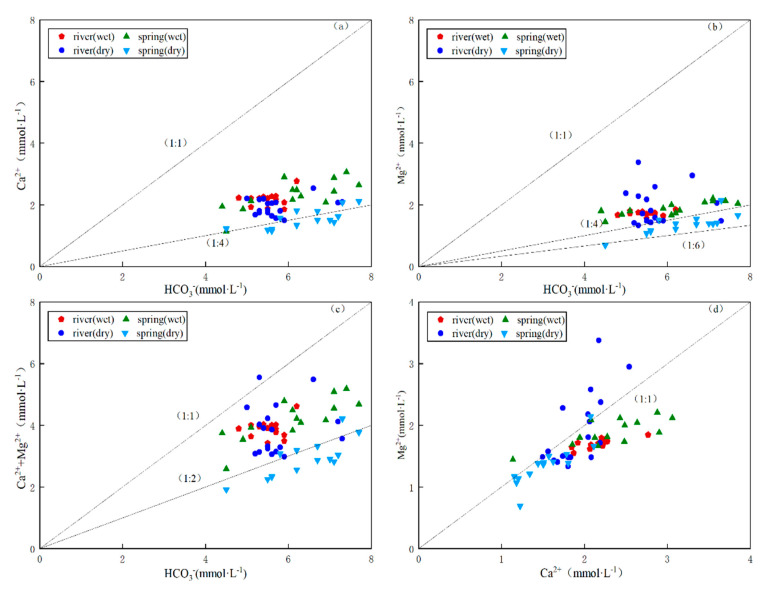
Proportion diagrams of ions with binary hydrogeochemical diagram. (**a**). Ca^2+^/HCO_3_^−^, (**b**). Mg^2+^/HCO_3_^−^, (**c**) (Mg^2+^ + Ca^2+^) /HCO_3_^−^ and (**d**) Mg^2+^/Ca^2+^.

In Figure 6a, γHCO_3_^−^ and γCa^2+^ ratios are on either side of the 1:4 line, and γHCO_3_^−^ and γMg^2+^ ratios are below 1:1 and above 1:6, respectively, in Figure 6b, indicating that one of the Ca^2+^ sources is carbonate rock. The bias is toward the 1:2 equivalence in the γ(Ca^2+^ + Mg^2+^) to γHCO_3_^−^ ratio in Figure 6c. A ratio near 1:1 for γCa^2+^ to γMg^2+^ in Figure 6d suggests that the weathered carbonate rock is predominantly dolomite in the study area.

### 4.2. Water Chemistry Evolutionary Processes

**Figure 7:**
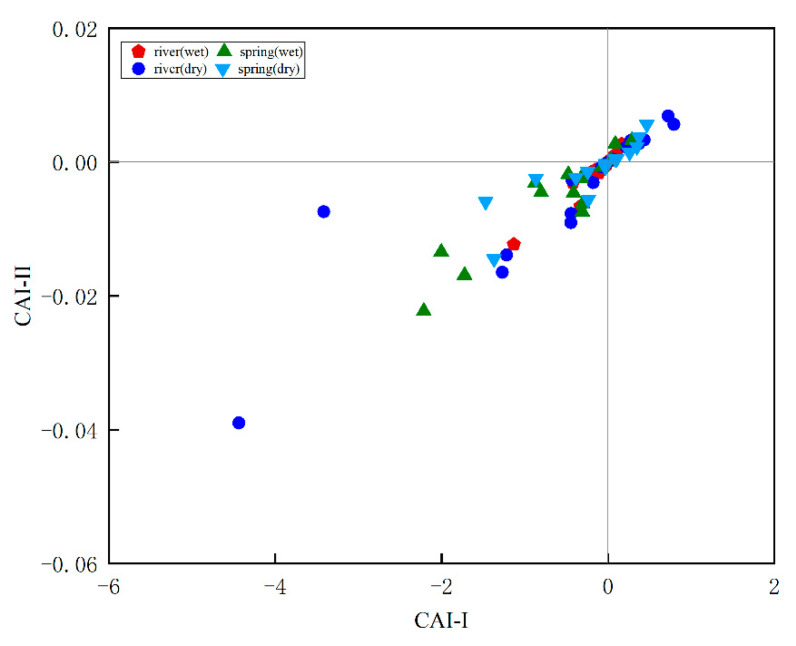
Diagram of chloro-alkaline indices.

Carbonate rocks contain anions on the surfaces of mineral particles, which, under certain conditions, release certain cations from the adsorbed surface and absorb new cations, which is called cation exchange. In general, the Na^+^ and K^+^ contents are relatively low in karst areas, being mainly produced from silicate rocks and evaporite rocks, where evaporation of water or ion exchange with clay minerals can also increase the K^+^ and Na^+^ contents in water [40]. Sodium–calcium exchange can change the concentration of cations in groundwater, an important process in the evolution of water chemistry [41]. The chlor-alkali index (CAI-I and CAI-II) was proposed by Schoeller [42]. If the values of both indices are negative, a certain amount of Ca^2+^ is replaced by a corresponding amount of Na^+^ in the soil. If the indices are positive, the reaction is occurring in the opposite direction. If a positive exchange occurs between Na^+^ and K^+^ in the groundwater and Ca^2+^ and Mg^2+^ in the aquifer, then the index is positive. In Figure 7, the distribution of the water samples shows that a positive exchange as well as a reverse exchange occur, so Ca^2+^ and Mg^2+^ are replaced by Na^+^ and K^+^ in the groundwater, explaining why some of the water samples in Figure 5 have a ratio greater than 1 and some have a ratio of less than 1.

(1) $$\mathrm{CAI-I}=\frac{{\mathrm{Cl}}^{-}-({\mathrm{Na}}^{+}+K^{+})}{{\mathrm{Cl}}^{-}}$$

(2) $$\mathrm{CAI-II}=\frac{{\mathrm{Cl}}^{-}-({\mathrm{Na}}^{+}+K^{+})}{{\mathrm{HCO}}_{3}^{-}+{\mathrm{SO}}_{4}^{2-}+{\mathrm{CO}}_{3}^{2-}+{\mathrm{NO}}_{3}^{-}}$$

**Figure 8:**
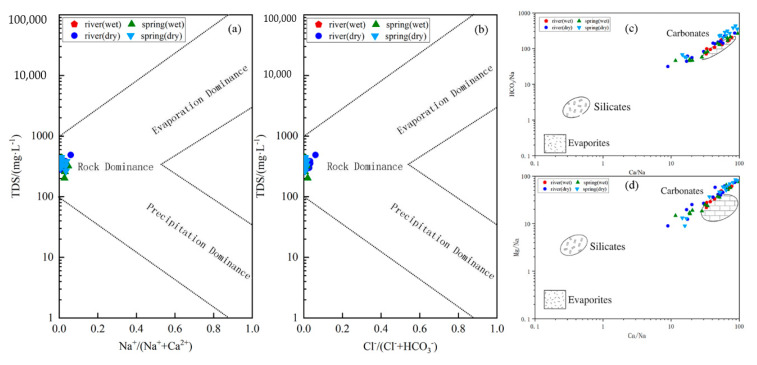
(**a**,**b**) Gibbs plots, (**c**) scatter diagram of Mg/Na vs. Ca/Na, and (**d**) scatter diagram of HCO_3_/Na vs. Ca/Na.

Gibbs diagrams are often used to determine the main controls of the evolution of water chemistry (evaporative crystallization, rock weathering, and atmospheric precipitation effects). The use of ratio plots of ions helps to consider the extent to which seawater, carbonate, and silicate rocks contributed to karst water ion concentrations [5,43]. In the Gibbs diagram, the lower right-hand corner of the diagram is dominated by atmospheric precipitation; in the middle left-hand part of the diagram, such sampling sites are mainly controlled by rock weathering; in the upper right-hand corner of the diagram, such streams are mainly controlled by evaporative crystallization. The scatter diagram is used to determine the main controls (carbonate, silicate, or evaporite) on the weathering process of the rocks. We found that the Huangzhouhe River basin is mainly controlled by rock weathering (Figure 8a,b), while the water samples are distributed in the carbonate rock(Figure 8c,d), indicating that the ionic composition of the water is mainly due to the weathering process of the rocks and is influenced by the weathering of carbonate rocks.

### 4.3. Impacts of Human Activities on Water Chemistry and Carbon and Nitrogen Contents

**Figure 9:**
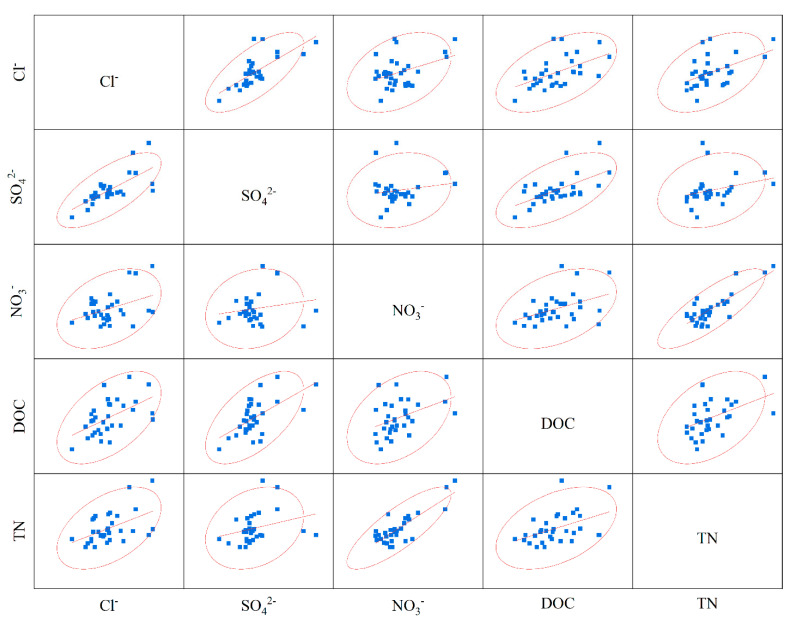
C, N, and ion correlation.

River water chemistry is controlled both by natural geochemistry and by anthropogenic disturbances. It is well known that river ions Cl^−^, SO_4_^2−^, and DIN are closely related to anthropogenic inputs (both agricultural and industrial) [8,44]. The overall sources of ions can be divided into atmospheric precipitation, rock weathering, and external inputs. The sources of NO_3_^−^ and SO_4_^2−^ are mainly rock weathering and anthropogenic inputs, Cl^−^, and atmospheric inputs. The watershed is mainly subject to the weathering of carbonate rocks. To estimate the contribution of rainfall, the lowest concentrations of Cl^−^ (0.73 mg·L^−1^) and NO_3_^−^ (1.2 mg·L^−1^) in the pristine area were assumed to represent atmospheric inputs [45]. The calculations show that the contribution of atmospheric inputs to Cl^−^ and NO_3_^−^ is less than 5% of the total dissolved ions. A positive correlation between DOC and Cl^−^ and SO_4_^2−^ is clearly shown in Figure 9, indicating anthropogenic pollution from the source.

Biological mechanisms of, e.g., phytoplankton and bacteria are considered to be important sources of river DOC [12]. The temporal and spatial changes in river DOC are largely affected by its source. In winter, the average DOC of river water is 3.33 mg·L^−1^ in the upper reaches and 2.90 mg·L^−1^ in the middle reaches. The average downstream DOC is 2.79 mg·L^−1^. In summer, the average DOC of the river is 3.37 mg·L^−1^, 3.37 mg·L^−1^, and 2.86 mg·L^−1^ in the upstream, middle, and downstream areas, respectively, decreasing along the river. The upstream agriculture is mainly rice and tobacco, and there are more phytoplankton in rice. The numbers of villages and farms increase from sampling points 1 to 10. Domestic sewage is discharged at will, and river water sampling points 9 and 10 are located in towns. Therefore, we found that the DOC is higher upstream. In summer, agricultural and domestic sewage have a greater impact, resulting in more nutrients in the water and greater phytoplankton activity. This led to higher DOC concentrations than in the dry season, but not higher than the world average level of 5.35 mg·L^−1^ [46], indicating that the Huangzhouhe River is less affected by human activities. With increasing distance from towns and villages, the population density in the middle reaches of the basin decreases, the farmland area decreases, and human pollution decreases. The downstream enters the core protected area, where humans have even less influence. With the gradual reduction in human influence, the river also has the ability to purify itself, so the DOC gradually decreases in the direction of the river. In winter, the average DOC in the upper, middle, and lower spring reaches is 1.75 mg·L^−1^, 3.09 mg·L^−1^, and 1.70 mg·L^−1^, respectively. In summer, the average upstream, midstream, and downstream DOC is 2.11 mg·L^−1^, 3.36 mg·L^−1^, and 1.75 mg·L^−1^, respectively. In karst areas, spring water is one of the water sources used in agriculture. Part of the spring water in the middle reaches of the river is used on rice fields. At the same time, there are aquatic plants in the spring water. This may be the reason for the high DOC in the spring water in the middle reaches. The upstream and midstream DICs in the watershed remain at the same level due to the influence of domestic sewage. The highest DIC in domestic sewage reaches 7397 µmol·L^−1^, having less of an effect in downstream areas [47,48].

In some river systems such as the Lichunhe and Daguhe Rivers in China, where DIN is the dominant form of N [36], TN is highly significantly and positively correlated with NO_3_-N (r = 0.96, *p* < 0.01; Table 3). NO_3_-N is the main component of DIN, a key nutrient essential for phytoplankton, and its main sources are biological and surface runoff. Changes in NO_3_-N lead to changes in TN, which may be due to the preferential uptake and use of DIN by phytoplankton and bacteria [49,50]. River NO_3_-N and TN contents are higher in the middle reaches, where anthropogenic influences are stronger. Downstream in the core protected area, anthropogenic influences are weaker and the self-purification capacity of the river results in decreased levels of NO_3_-N and TN. Spring NO_3_-N and TN contents are the same in the upper stream and midstream and lower in the lower reaches, probably due to a decrease in anthropogenic influences.

Within the Huangzhouhe River Basin, DOC levels are higher in rivers than in springs, and TN and NO_3_-N levels are higher in springs than in rivers, mainly because plankton consume DIN to release DOC and because of anthropogenic pollution. Spring DIC levels are significantly higher than those in river water due to the longer duration of water–rock action in springs. The trend in carbon and nitrogen in springs is consistent with that of river water, indicating that springs and river water are independent, interacting, and interdependent in the hydrological system. Compared with river water, spring water is less affected by the outside world. TN and NO_3_-N are extremely significantly positively correlated (r = 0.96, *p* < 0.01; Table 3), with roughly the same range of values, with NO_3_-N accounting for as high as 83% of the TN. This shows that the output of carbon and nitrogen in the Huangzhouhe River Basin is mainly DIC and NO_3_-N.

### 4.4. Relationship between C and N

A total of 38 species of algae in the Huangzhouhe River Basin were detected in 6 phyla, 29 families, 37 genera, 5 phyla, 25 genera, and 25 species in summer; 3 phyla, 17 genera, and 18 species in winter. Diatom species are dominant. In summer, diatoms and green algae are positively correlated with NO_3_-N; in winter, oscillating algae are mainly negatively correlated with TN and NH_3_-N, and *Scenedesmus dimorpha* in the phylum Chlorophyta is positively correlated with NH_3_-N [51].

The relationship between water pH and carbon-to-nitrogen ratio is complex: aquatic plants and animals consume relative levels of NO_3_^−^ and NH_4_^+^ and thus have a direct role in regulating water pH, and river C:N has an important influence on the growth and reproduction of plankton in water [52,53]. Planktonic algae and aquatic plants can absorb and utilize water DIC through photosynthesis [54], so lower levels of planktonic algae lead to weaker photosynthesis in the water column, which, in turn, reduces the amount of DIC absorbed and used in the water [55]. These processes inevitably cause changes in the pH of the water [56], which affect the growth activities of microorganisms and algae [57,58], which affect DOC release and TN decomposition. Dead cells of planktonic algae release high-molecular-weight DOC, while living cells release low-molecular-weight DOC [59]. DOC has an important role in the process of biological denitrification and denitrification [60], where the organic carbon source acts both as an energy source for denitrifying bacteria to synthesize cells and as an electron donor in the denitrification reaction, which can facilitate the conversion of nitrogen in the water column into N_2_O and N_2_ to be released from the water column into the atmosphere. This reduces the TN content of the water column. There is a correlation between TN and DOC (r = 0.42, *p* < 0.05; Table 3). Therefore, microorganisms may be the main factor causing carbon and nitrogen coupling in conventional water bodies in the basin. Anthropogenic activities have an impact on water chemistry characteristics, affecting phytoplankton, which, in turn, affects the carbon and nitrogen contents, which, in turn, affect the water chemistry characteristics.

According to the stoichiometric ratio of nutrients in the plant plankton body proposed by Redfield [61], C:N:P = 106:16:1, it can be seen that the C:N suitable for the growth of plankton in the basin water should be about 6.6 The C:N range of the conventional water body of the Huangzhouhe River is 1.7–4.66, with an average of 3.25. Although it has not reached the optimal growth state, good living conditions are provided for plankton, indicating that the pollution caused by human activities is relatively low in the Huangzhouhe River and that the Huangzhouhe River ecosystem is healthy.

### 4.5. Water Quality Assessments

Based on the Sanitary Standards for Drinking Water (GB 5749-2006) [62], some of the water quality parameters (pH, Cl^−^, NO_3_−N, SO_4_^2^^−^, and TDS) are shown in Table 4. The content of N species in the water samples in the basin is within the standard range, and the DOC content is far less than the average level of 5.35mg·L^−1^ in the world’s rivers. Shibing is a World Natural Heritage Site, with no serious industrial activities, and only a small area used for traditional agricultural activities. Large areas of virgin forest and diversified ecosystems have been protected and developed, indicating that the world’s natural heritage is being protected.

## 5. Conclusions

In this study, we found that river and spring water samples within the Huangzhouhe River Basin are dominated by Ca^2+^, Mg^2+^, and HCO_3_^−^. Ca^2+^ and Mg^2+^ account for 97% of the total cation concentration. For anions, HCO_3_^−^ accounts for about 96% of the total anion concentration. The higher HCO_3_^−^ content in the spring water than in the river water is mainly due to the longer duration of spring water–rock action. The water chemistry identified by the Piper diagram is dominated by HCO_3_-Ca-Mg. Through ion correlation, we found that the correlation between Cl^−^ and SO_4_^2−^ in river water is stronger than that in spring water, which is due to human influences. The ion ratios, Gibbs plots, and scatter diagram reveal that the water chemistry of the Huangzhouhe River Basin is mainly influenced by the weathering of dolomite rocks, and that cation exchange is also present.

Within the Huangzhouhe River Basin river water, the DOC content decreases gradually in the river direction, mainly due to decreases in population density and anthropogenic impacts. The spring water DOC content is higher in the middle reaches, mainly due to its use for agriculture with more aquatic plants. The river and spring water DIC contents increase in midstream areas, which may be due to inputs of domestic wastewater. Due to the impact of agricultural and domestic sewage, the DOC content increases with increases in Cl^−^ and SO_4_^2−^. The TN and NO_3_-N contents in rivers change with the change in population density, reaching a maximum in the middle reaches; the TN and NO_3_-N contents in spring water are lower downstream. The trends of the changes of the river and spring water contents are the same, with a higher DOC content in river water than in river water, and NO_3_-N leading to less TN in river water than in spring water, mainly due to the reduction in consumption by plants and organisms’ activities in the river. pH and water temperature affect phytoplankton and bacterial activity, and phytoplankton affect C and N concentrations, so microorganisms may be causing carbon and nitrogen coupling in the conventional waters of the basin. The main carbon and nitrogen species in the Huangzhouhe River Basin are HCO_3_^−^ and NO_3_^−^, respectively.

The water quality index content of the Huangzhouhe River Basin complies with Sanitary Standards for Drinking Water (GB5749-2006). Although the Huangzhouhe River is subject to tourism and agricultural impacts, the water quality is still good.

## Figures and Tables

**Table 1:** Statistics of the hydrochemical parameters of the Huangzhouhe River Basin (mg·L^−1^).

**Dry**	**pH**	**Ca^2+^**	**Mg^2+^**	**K^+^**	**Na^+^**	**Cl^−^**	**NO_3_^−^**	**SO_4_^2^^−^**	**HCO_3_^−^**	**TDS**
Minimum	6.98	46.15	16.56	0.11	0.08	0.43	1.20	4.63	274.50	265.60
Maximum	8.19	101.57	80.94	5.13	5.29	5.79	8.51	28.53	469.70	488.50
Average	7.46	70.25	40.12	1.40	1.10	2.27	3.68	16.19	367	348.58
SD	0.29	13.69	13.30	1.26	1.11	1.31	1.99	6.05	48.71	51.01
**Wet**	**pH**	**Ca^2+^**	**Mg^2+^**	**K^+^**	**Na^+^**	**Cl^−^**	**NO_3_^−^**	**SO_4_^2−^**	**HCO_3_^−^**	**TDS**
Minimum	7.19	45.47	34.66	0.49	0.55	0.92	2.61	8.84	268.4	202.30
Maximum	8.99	122.40	52.93	3.37	2.59	5.45	26.64	26.08	469.7	440.20
Average	8.16	89.71	43.10	1.52	1.31	2.60	8.84	17.12	355.3	338.40
SD	0.48	15.54	4.40	0.87	0.62	1.20	7.67	5.44	51.53	54.69

**Table 2:** C and N species in the Huangzhouhe River Basin (mg·L^−1^).

	Parameter	Upper Huangzhouhe		Middle Huangzhouhe		Lower Huangzhouhe	
		Range	Mean Value	Range	Mean Value	Range	Mean Value
River (dry)	DOC	2.46–4.52	3.33	2.34–3.77	2.90	2.04–3.54	2.79
	DIC	305–445.3	351.36	317.2–439.2	356.85	323.3–329.4	326.35
	NO_3_-N	0.49–0.79	0.63	0.76–1.19	0.92	0.64–0.7	0.67
	TN	0.62–1.33	0.9	1.04–1.91	1.26	0.89–0.93	0.91
River (wet)	DOC	2.57–4.81	3.38	2.82–3.96	3.37	2.86	2.86
	DIC	323.3–378.2	347.7	311.1–347.7	324.83	292.87	292.8
	NO_3_-N	0.85–1.99	1.20	0.77–1.29	1.01	0.90	0.90
	TN	1.22–3.23	1.89	1.29–1.97	1.54	1.06	1.06
Spring (dry)	DOC	1.56–1.93	1.75	1.77–4.85	3.09	1.48–1.8	1.70
	DIC	378.2–433.1	405.65	335.5–469.7	407.16	274.5–408.7	341.6
	NO_3_-N	0.15–1.92	1.04	0.27–1.4	1	0.7–0.88	0.81
	TN	0.59–2.99	1.44	0.62–2.63	1.50	0.83–1.55	1.17
Spring (wet)	DOC	1.90–2.33	2.11	2.11–4.23	3.36	1.66–1.86	1.75
	DIC	420.9–433.1	427	274.5–469.7	390.4	268.4–372.1	312.63
	NO_3_-N	0.59–1.55	1.07	0.89–6.01	3.54	1.15–4.75	2.43
	TN	1.84–2.16	2	1.62–3.78	2.97	1.99–3.32	2.70

DIC: dissolved inorganic carbon; DOC: dissolved organic carbon;NO_3_-N: nitrate; TN: dissolved organic nitrogen.

**Table 3:** Pearson correlation coefficients between C and N species and physicochemical parameters in the Huangzhouhe River Basin.

Parameters	T	PH	EC	TDS	DIC	DOC	NO_3_-N	TN
T	1							
pH	0.83 **	1						
EC	0.46 *	−0.31	1					
TDS	−0.17	−0.32	0.36 *	1				
DIC	−0.17	−0.10	0.71	0.58 **	1			
DOC	0.20	0.77	0.32	0.35 *	−0.08	1		
NO_3_-N	0.64 **	0.33	0.61 **	0.29	0.003	0.39 *	1	
TN	0.67 **	0.42	0.56 **	0.27	0.14	0.42 *	0.96 **	1

*, ** Significance at 0.05 and 0.01 probability levels, respectively. T: temperature; EC: conductivity; TDS: total dissolved solids.

**Table 4:** Water quality determination (mg·L^−1^).

Parameter	pH	Cl^−^	NO_3_^−^N	SO_4_^2−^	TDS
Standard range	6.5-8.5	<250	<10	<250	<1000
Water sample	6.98–8.99	0.43–5.79	0.15–3.05	4.62–28.53	202.3–488.5
Unqualified	1	0	0	0	0

## Data Availability

The data presented in this study are available on request from the corresponding author.
